# Paroxysmal sympathetic hyperactivity syndrome caused by fat embolism syndrome

**DOI:** 10.5935/0103-507X.20180035

**Published:** 2018

**Authors:** Daniel Agustin Godoy, Jose Orquera, Alejandro A. Rabinstein

**Affiliations:** 1 Unidade de Terapia Neurointensiva, Sanatório Pasteur - Catamarca, Argentina.; 2 Unidade de Terapia Intensiva, Hospital San Juan Bautista - Catamarca, Argentina.; 3 Pronto-Socorro, Hospital San Juan Bautista - Catamarca, Argentina.; 4 Unidade de Terapia Intensiva de Neurociência, Mayo Clinic - Rochester, Minnesota, Estados Unidos.

**Keywords:** Autonomic nervous system diseases, Primary dysautonomia, Brain injuries, Embolism, fat

## Abstract

Paroxysmal sympathetic hyperactivity represents an uncommon and potentially life-threatening complication of severe brain injuries, which are most commonly traumatic. This syndrome is a clinical diagnosis based on the recurrent occurrence of tachycardia, hypertension, diaphoresis, tachypnea, and occasionally high fever and dystonic postures. The episodes may be induced by stimulation or may occur spontaneously. Underdiagnosis is common, and delayed recognition may increase morbidity and long-term disability. Trigger avoidance and pharmacological therapy can be very successful in controlling this complication. Fat embolism syndrome is a rare but serious complication of long bone fractures. Neurologic signs, petechial hemorrhages and acute respiratory failure constitute the characteristic presenting triad. The term cerebral fat embolism is used when the neurological involvement predominates. The diagnosis is clinical, but specific neuroimaging findings can be supportive. The neurologic manifestations include different degrees of alteration of consciousness, focal deficits or seizures. Management is supportive, but good outcomes are possible even in cases with very severe presentation. We report two cases of paroxysmal sympathetic hyperactivity after cerebral fat embolism, which is a very uncommon association.

## INTRODUCTION

Paroxysmal sympathetic hyperactivity (PSH) is a complication that may increase the morbidity and mortality of neurocritically ill patients, especially if it is not promptly recognized and treated.^(^^1^^-^^5^^)^ It is characterized by the sudden appearance of signs and symptoms caused by increased sympathetic discharge. Hypertension, tachycardia, tachypnea, fever, profuse sweating and dystonic postures constitute the classic clinical picture.^(^^1^^-^^5^^)^ Diagnostic criteria have been proposed, although their validation is pending.^(^^6^^)^ Severe traumatic brain injury is the most frequent cause of PSH,^(^^4^^)^ although other acute neurological insults can also result in this complication.^(^^1^^,^^2^^)^ A very uncommon cause of PSH is fat embolism syndrome (FES). Fat embolism syndrome occurs due to the massive passage of fat particles from the bone marrow into the systemic circulation as a result of fractures of long bones or pelvic bones or as a consequence of surgical intramedullary fixation procedures.^(^^7^^-^^9^^)^ The typical features of FES are acute respiratory distress, petechial rash and various neurological manifestations.^(^^7^^-^^9^^)^ When neurological signs and symptoms predominate, the condition is often referred to as cerebral fat embolism.^(^^10^^-^^13^^)^ In such cases, brain magnetic resonance imaging (MRI) is very useful to support the diagnosis.^(^^14^^,^^15^^)^

Herein, we present two cases of FES manifesting with PSH and hypothesize about the underlying pathophysiology of this rare clinical association.

## CASE REPORTS

### Case 1

**Figure 1:**
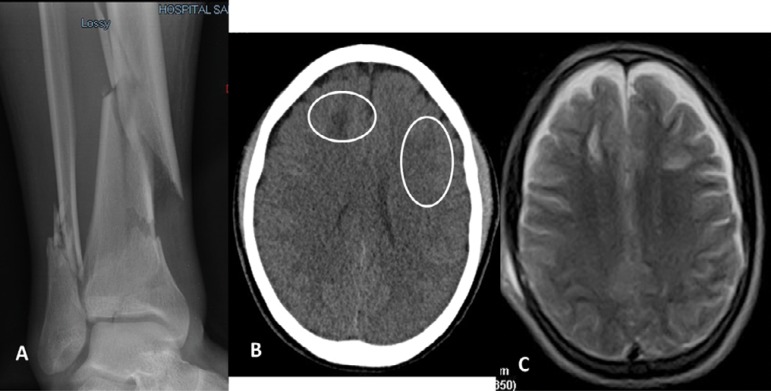
Images of patient 1 at admission to the emergency room. (A) Multi-fragmentary fractures of the lower third of right tibia and fibula. (B) Computed tomography scan showing bilateral frontal subcortical hypodense areas without a midline shift. No hemorrhage was evident, and the basal cisterns and sulcus remained visible. (C) Magnetic resonance image showing multiple lesions in both cerebral hemispheres that were hypointense on T1 and hyperintense on T2 and FLAIR sequences in the periventricular white matter of both frontoparietal regions.

A previously healthy 25-year-old man presented with multi-fragmentary fractures of the lower third of right tibia and fibula as a consequence of a motorcycle accident (Figure 1A). Upon hospital admission he was alert, coherent and had no motor deficits. His vital signs and the rest of a physical examination were normal. He was admitted to the hospital for surgical stabilization. Forty-eight hours after admission, he developed confusion and agitation followed by a rapid decline in his level of consciousness that progressed to coma with bilateral extensor posturing. His pupils were equal, slightly large and reactive. He was tachypneic (44/min), tachycardic (137/min), febrile (39.3°C) and hypertensive (147/101mmHg). His pulse oximetry was 92% on room air. Petechial hemorrhages were noted in the sclerae, conjunctivae, buccal mucosa and the upper third of the thorax. Resuscitation was initiated with fluids, supplemental oxygen, tracheal intubation and mechanical ventilation under deep sedoanalgesia. A head computed tomography (CT) scan revealed multiple and bilateral frontal subcortical hypodense areas without a midline shift. No hemorrhage was evident, and the basal cisterns and sulci remained visible (Figure 1B).

A chest CT was normal except for small bilateral basal atelectasis with no evidence of pulmonary embolism. Transthoracic echocardiography revealed normal ventricles, normal valve function and an absence of patent foramen oval or signs of pulmonary hypertension. An electroencephalogram revealed diffuse slowing without epileptiform discharges. The laboratory parameters were normal except for elevations of the following inflammation markers: leukocytosis 17300/mm^3^ and C-reactive protein (CRP) 141mg/L. Thrombocytopenia (110.000/mm^3^) was also noticed. A diagnosis of FES was reached. Supportive therapy was provided, and methylprednisolone (10mg/kg/day) was administered for 3 days. Two days later, given his clinical stability, the sedation and analgesia were withheld to assess his neurological status. After 10 minutes, bilateral and spontaneous extensor posturing was observed in combination with mechanical ventilation asynchrony, tachypnea (46/min), profuse and generalized sweating, arterial hypertension (215/112mmHg) and tachycardia (137/min). These manifestations were recurrent and led to the diagnosis of PSH. Consequently, propranolol (120mg/d) and morphine (3mg) every 4 hours were added to the therapeutic regimen. The sedoanalgesia was reinstituted. Two days later, a brain MRI revealed multiple small lesions in both cerebral hemispheres that were hypointense on T1 and hyperintense on T2 and FLAIR, did not exhibit enhancement after gadolinium injection and were located in the periventricular white matter of both frontoparietal regions (Figure 1C).

The episodes of PSH had durations that oscillated between 20 and 35 minutes, occurred with a frequency of 3 to 4 times daily and were mainly associated with stimuli such as the turning, bathing, aspiration of secretions and pain. On the 8^th^ intensive care unit (ICU) day, remifentanil and propofol were withdrawn and replaced with dexmedetomidine while the propranolol and morphine were maintained at the previous doses. The episodes of sympathetic discharge became progressively briefer and less severe and reached no more than 2 per day. The motor responses improved to the localization of the nociceptive stimulus.

On ICU day 12, the patient was extubated. Sixteen days after admission, he was alert, had normal motor responses and was able to communicate with his family. The doses of propranolol and morphine were reduced to half without worsening of the episodes of PSH. Twenty-two days after admission, the fracture was surgically fixed. The post-operative course proceeded without complications. Three days after surgery, the patient was discharged home to continue ambulatory rehabilitation. One year after the event, the lesions found in the MRI had disappeared, and neuropsychological testing revealed a mild impairment of executive functions with alterations of short and long-term memory.

### Case 2

**Figure 2:**
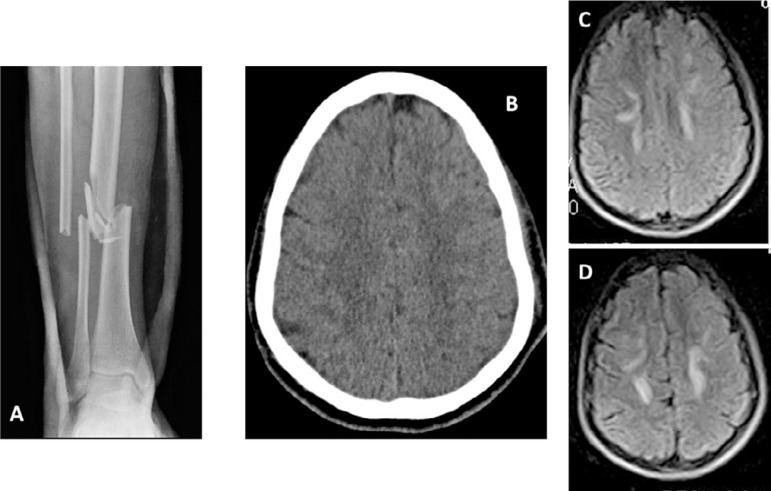
Patient 2 images at admission. (A) Closed fracture of the lower third of the tibia. (B) Computed tomography scan showing subcortical hypodense lesions in the frontal and left parietal regions. (C-D) Magnetic resonance images showing multiple T2-hyperintense lesions localized in periventricular white matter and bilateral frontal and parietal subcortical regions.

A 21-year-old man without any medical history presented with a closed fracture of the lower third of his tibia secondary to a motorcycle accident without evidence of cranial trauma (Figure 2A). His vital signs and neurological examination were normal. The patient was hospitalized and treated with skeletal traction. One day later, he became confused and agitated with progressive depression of consciousness that progressed to coma. He exhibited an extensor motor response, but the brainstem reflexes were normal. The vital signs were as follows: arterial blood pressure, 127/76mmHg; respiratory rate, 18/min; heart rate, 133/min; and rectal temperature, 38.8°C. His pulse oximetry was 85% on room air. Invasive mechanical ventilation and hemodynamic resuscitation were initiated immediately. After cardiorespiratory stabilization, a head CT scan revealed small and multiple subcortical hypodense lesions in both the frontal and left parietal regions (Figure 2B).

A chest CT revealed small filling defects in the left ventricle and the superior vena cava. A nodular image was observed in the right pulmonary artery and could have corresponded to an embolus. Fat embolism syndrome was diagnosed. A transthoracic echocardiogram revealed an estimated mean pulmonary artery pressure of 35mmHg with no disorders of ventricular motility and preserved systolic ventricular function. The size and function of the right ventricle were normal, and there were no interatrial or interventricular shunts. An electroencephalogram revealed background slowing without epileptiform discharges. The biochemical profile was unremarkable with the exception of thrombocytopenia (98.000/mm^3^), anemia (Hgb 8.7gr/dL) and an elevation of the CRP level to 134 mg/L. General critical care supportive measures were provided, and the patient received IV methylprednisolone at 10 mg/kg/day for 72 hours.

On ICU day 5, sedoanalgesia (remifentanil-propofol) was stopped. After 20 minutes, the patient exhibited generalized and profuse sweating, tachycardia (166/min), arterial hypertension (187/99mmHg), and tachypnea (36/min) with asynchrony of mechanical ventilation and bilateral extensor posturing; these manifestations were all compatible with PSH. The symptoms were alleviated with the restoration of sedoanalgesia but continued to occur 4 times per day and were triggered by physiotherapy maneuvers, aspiration of secretions and baths with cold water. The average duration of the episodes was 33 minutes. A brain MRI revealed multiple T2-hyperintense lesions in the periventricular white matter and bilateral frontal and parietal subcortical regions (Figure 2C and D).

Treatment with propranolol at 120mg/day, morphine at 18mg/day and gabapentin at 900 mg/day resulted in progressive reductions of the frequencies, intensities and durations of the PSH episodes. The patient was extubated without complications on hospital day 11. After 20 days, he was transferred to the general ward; he was alert, oriented, communicating with his family and feeding on his own. He was maintained on propranolol at 40mg/day and morphine at 6mg/day. Surgical fixation of the fracture was performed one month after admission. He was discharged to home three days after the surgery. At 12 months after the event, the previously noted lesions were no longer visible on a follow up MRI, and the patient returned to his work and his classes at the university without alterations in neurocognitive testing results.

## DISCUSSION

We presented two cases of FES with severe cerebral involvement that developed PSH as a complication. A high level of awareness of this complication and adequate sedation holidays allow for the early detection and treatment of PSH. Despite the dramatic neurological presentation (coma and extensor posturing), both patients recovered well with aggressive supportive management and pharmacological control of the PSH.

We are only aware of two previous cases of FES complicated with PSH.^(^^16^^,^^17^^)^ Although the association is rare, we are confident of the two diagnoses in our patients. Both patients met Gurd's clinical and radiological criteria for FES (Table 1)^(^^18^^)^ and had high probability scores for PSH^(^^6^^)^ (20 and 21 for the first and second cases reported, respectively; Table 2). The pathophysiology of PSH has not been elucidated, but it has been postulated that a disconnection between the cortical and diencephalic inhibitory centers within the brainstem and the spinal cord could play a central role.^(^^5^^)^ We postulate that the embolic shower in FES may affect the cerebral areas responsible for modulating central sympathetic outflow and thus cause PSH.

**Table 1:** Original and modified Gurd’s criteria for fat embolism syndrome diagnosis

Gurd and Wilson criteria*	Modified criteria#
Major	
Hypoxemia	PaO_2_ < 60mmHg at FiO_2_ 0.21 with or without pulmonary infiltrate on X-ray
Altered mentality	Altered mentality with multiple cerebral white matter lesion in MRI
Petechiae	Petechiae on conjunctiva and upper trunk
Minor	
Tachycardia	Heart rate > 100/min
Fever	Temperature > 38°C
Thrombocytopenia	Platelets < 100 x 10^3^/µL
Unexplained anemia	Anemia with coagulopathy or DIC without bleeding site
Anuria or oliguria	Anuria or oliguria
Retinal embolism	Retinal embolism
Fat globule in urine or sputum	Not included
Jaundice	Not included
High ESR	Not included

* Two major criteria or 1 major + 4 minor criteria are required for diagnosis.^(^^21^^)^

# One major + 3 minor or 2 major + 2 minor criteria are required for diagnosis.^(^^18^^)^ PaO_2_ - partial pressure of oxygen; FiO_2_ – fraction of inspired oxygen ; MRI - magnetic resonance imaging; DIC - disseminated intravascular coagulation; ESR - erythrocyte sedimentation rate.

**Table 2:** Paroxysmal sympathetic hyperactivity diagnostic likelihood tool^(^^6^^)^

	Clinical feature scale						Score	
	0		1	2		3		
Heart rate	< 100		100 - 119	120 -139		> 140		
Respiratory rate	< 18		18 - 23	24 - 29		> 30		
Systolic blood pressure	< 140		140 - 159	160 - 179		> 180		
Temperature	< 37		37 - 37.9	38 - 38.9		> 39		
Sweating	Nil		Mild	Moderate		Severe		
Posturing during episodes	Nil		Mild	Moderate		Severe		
						CFS Subtotal		
**Severity of clinical features**		Nil			0			
		Mild			1 - 6			
		Moderate			7 - 12			
		Severe			≥ 13			
**DLT**								
Clinical features occur simultaneously								
Episodes are paroxysmal in nature								
Sympathetic over-reactivity to normally non-painful stimuli								
Features persist ≥ 3 consecutive days								
Features persist ≥ 2 weeks post brain injury								
Features persist despite treatment of alternative differential diagnoses								
Medication administered to decrease sympathetic features								
≥ 2 episodes daily								
Absence of parasympathetic features during episodes								
Absence of other presumed cause of features								
Antecedent of acquired brain injury								
(Scored 1 point for each feature present)								
**DLT subtotal**								
**Combined total (CFS + DLT) **								
**PSH diagnostic likelihood**		Unlikely			< 8			
		Possible			8 - 16			
		Probable			> 17			

CFS - clinical feature scale; DLT - diagnosis likelihood tool; PSH - paroxysmal sympathetic hyperactivity.

### Fat embolism syndrome

Echocardiographic and Doppler studies have demonstrated that ≥the passage of fat particles into the circulation system is a frequent phenomenon after orthopedic surgery or long bone fracture; however, this situation is not sufficient to trigger FES.^(^^17^^,^^19^^,^^20^^)^ The reported incidence of FES varies from 0.5 to 29% depending on the study type (clinical or pathological).^(^^7^^-^^9^^,^^21^^)^ FES mainly affects the young adult population with a mean of 30 years of age.^(^^7^^-^^9^^)^ The fracture of long bones (e.g., the femur, tibia, and pelvis), orthopedic surgery (intramedullary fixation and arthroplasty), liposuction, severe burns and bone marrow transplantation are the entities that most frequently predispose patients to the development of the syndrome.^(^^7^^-^^9^^)^

The classic clinical picture consists of the gradual appearance (24 - 72 hours post-injury) of signs and symptoms caused by pulmonary, cerebral and skin involvement; however, this triad only appears simultaneously in approximately half of the cases.^(^^7^^-^^9^^)^ The diagnostic criteria are presented in table 1. Neurological signs and symptoms are present in 80 - 85% of cases,^(^^6^^-^^8^^)^ but the incidence of cerebral fat embolism (i.e., a fat embolism presenting with predominant neurological manifestations) is unknown.^(^^10^^-^^13^^)^ The main neurological feature of FES is an alteration of consciousness that can range from confusion to coma, and few more than 20% of cases may present with focal deficits, abnormal motor responses or seizures.^(^^12^^,^^13^^,^^17^^)^ Brain MRIs can be very helpful to support the diagnosis by showing small multifocal lesions that are hypointense on T1-weighted sequences and hyperintense on T2-weighted sequences.^(^^14^^,^^15^^)^ These lesions are non-confluent, and they are preferentially located in the subcortical white matter in border zone areas between arterial territories, the centrum semiovale, basal ganglia, thalamus, brainstem and cerebellum.^(^^14^^,^^15^^)^ The ''starfield pattern'' is characteristic.^(^^14^^)^ The lesions can be observed during the hyperacute phase on diffusion-weighted imaging (DWI) sequences, and the hyperintensities on T2 sequences may take several days to become visible.^(^^15^^)^ Initial hypodense lesions correspond to microbleeds as corroborated in pathological studies.^(^^22^^)^

The treatment of FES is supportive.^(^^7^^-^^13^^,^^17^^)^ The use of corticosteroids is controversial and does not rely on solid scientific evidence.^(^^22^^)^ The mortality rate of FES is approximately 10%.^(^^23^^)^ Among the survivors who initially present with abnormal motor posturing or coma, 58% achieve good functional recovery. Intellectual sequelae may only become evident with formal neurocognitive testing.^(^^23^^)^

### Paroxysmal sympathetic hyperactivity

Inconsistent nomenclature has limited our understanding of this condition.^(^^1^^-^^5^^,^^24^^)^ Paroxysmal sympathetic hyperactivity has recently become the preferred term for this disorder.^(^^1^^-^^5^^)^ This syndrome involves a set of nonspecific signs and symptoms that denote exacerbated sympathetic discharge.^(^^1^^-^^5^^,^^25^^)^ The episodes start suddenly and are recurrent, but their duration, frequency, and severity can vary.^(^^1^^-^^5^^,^^24^^,^^25^^)^ Paroxysmal sympathetic hyperactivity is a common complication of severe traumatic brain injury, and in such cases, its incidence ranges from 15 - 33%.^(^^1^^-^^5^^,^^24^^)^ Paroxysmal sympathetic hyperactivity can also be observed after ischemic and hemorrhagic strokes, anoxic encephalopathy and encephalitis.^(^^1^^-^^5^^,^^24^^,^^25^^)^

The clinical picture is associated with elevated levels of circulating catecholamines and is characterized by features that indicate increased sympathetic activity, such as tachycardia, hyperthermia, tachypnea, arterial hypertension, generalized sweating, and abnormal and dystonic motor posturing, which can result in asynchrony with mechanical ventilation.^(^^1^^-^^4^^,^^24^^-^^27^^)^ In general, the clinical features appear simultaneously and abruptly either spontaneously or upon triggering by external stimuli (e.g., pain, baths, physiotherapy, aspiration of secretions, etc.).^(^^1^^-^^5^^,^^24^^,^^25^^)^ The mean duration of each episode is 30 minutes, and the mean frequency is 3 to 5 times a day.^(^^1^^-^^5^^,^^24^^,^^25^^)^ The diagnosis in the ICU is most commonly established between 5 and 7 days post-injury,^(^^1^^-^^5^^,^^24^^)^ but delayed diagnosis and underdiagnosis are not uncommon. A recent consensus of international experts proposed a scoring tool to quantify the diagnostic probability of PSH that relies on a scale that categorizes the presence and severity of the clinical components.^(^^6^^)^ High diagnostic probability is observed when the score is greater than 17 points^(^^6^^)^ (Table 2).

The treatment of PSH syndrome is based on avoiding stimuli than may trigger the episodes and administering drugs to abort the crises; these drugs principally include morphine and propranolol or clonidine.^(^^25^^,^^28^^-^^31^^)^ Simultaneously, prophylactic treatment of the episodes should be initiated. Gabapentin has been most useful in our experience, but bromocriptine, clonidine and baclofen have also been used with variable success.^(^^25^^,^^28^^-^^31^^)^ Gabapentin can be added to abortive therapy when hypertonicity predominates or when episodes of paroxysmal discharge persist despite the maximum dose of morphine and propranolol as reported in the second case. The doses used range from 300 to 4800mg/day.^(^^25^^,^^28^^-^^31^^)^ If not properly controlled, the syndrome can lead to serious complications, such as dehydration, muscle loss, severe contractures, aspiration, and even heart failure. A recent study demonstrated that 6 months after a severe head trauma, 61% of the patients who had developed PSH were dead, and another 30% were severely disabled.^(^^3^^)^ The prognoses are worse in patients with more severe manifestations of PSH.^(^^3^^)^ The degree to which PSH itself contributes to these poor outcomes remains to be elucidated.

## CONCLUSIONS

These cases illustrate a few useful clinical points. Fat embolism syndrome (occasionally with predominant cerebral manifestations) should be promptly suspected in patients who suffer neurological deterioration after a long bone fracture. In these cases, paroxysmal sympathetic hyperactivity can occur as a secondary complication that can be effectively treated, especially if adequate treatment is initiated early. Patients with fat embolism syndrome and paroxysmal sympathetic hyperactivity can become critically ill both neurologically and systemically; however, excellent recovery is possible even in very severe cases.
